# Demand Creation for Polio Vaccine in Persistently Poor-Performing Communities of Northern Nigeria: 2013–2014

**DOI:** 10.1093/infdis/jiv511

**Published:** 2016-04-02

**Authors:** Charity Warigon, Pascal Mkanda, Ado Muhammed, Andrew Etsano, Charles Korir, Samuel Bawa, Emmanuel Gali, Peter Nsubuga, Tesfaya B. Erbeto, George Gerlong, Richard Banda, Yared G. Yehualashet, Rui G. Vaz

**Affiliations:** 1 World Health Organization, Country Representative Office, and; 2 National Primary Health Care Development Agency, Abuja, Nigeria;; 3 World Health Organization, Regional Office for Africa, Brazzaville, Congo; and; 4 Global Public Health Solutions, Atlanta, Georgia

**Keywords:** unmet health needs, underserved communities, demand creation, noncompliance, persistently poor performing high risk communities, persistently poor performing LGAs, Northern Nigeria

## Abstract

***Introduction.*** Poliomyelitis remains a global threat despite availability of oral polio vaccine (OPV), proven to reduce the burden of the paralyzing disease. In Nigeria, children continue to miss the opportunity to be fully vaccinated, owing to factors such as unmet health needs and low uptake in security-compromised and underserved communities. We describe the implementation and evaluation of several activities to create demand for polio vaccination in persistently poor-performing local government areas (LGAs).

***Methods.*** We assessed the impact of various polio-related interventions, to measure the contribution of demand creation activities in 77 LGAs at very high risk for polio, located across 10 states in northern Nigeria. Interventions included provision of commodities along with the polio vaccine.

***Results.*** There was an increasing trend in the number of children reached by different demand creation interventions. A total of 4 819 847 children were vaccinated at health camps alone. There was a reduction in the number of wards in which >10% of children were missed by supplementary immunization activities due to noncompliance with vaccination recommendations, a rise in the proportion of children who received ≥4 OPV doses, and a decrease in the proportion of children who were underimmunized or unimmunized.

***Conclusions.*** Demand creation interventions increased the uptake of polio vaccines in persistently poor-performing high-risk communities in northern Nigeria during September 2013–November 2014.

Poliomyelitis has remained endemic in 3 countries in 2 regions of the World Health Organization (WHO), resulting in continued shifting of the target date for global eradication. By the end of 2011, polio remained a global threat despite the availability of oral polio vaccine (OPV), widely acclaimed as one of public health triumphs, given its success in reducing the health burden of the paralyzing disease. Because of the difficulty in eradicating polio, in May 2012 the World Health Assembly declared polio eradication as a programmatic public health emergency of global significance [1, 2].

In Nigeria, some children continued to miss the opportunity to be fully vaccinated because, for various reasons (eg, religious and sociocultural beliefs), parents and caregivers do not allow them to be fully vaccinated. In some instances, vaccination programs miss children because of compromised security and population mobility. These missed opportunities conflict with the Convention on the Rights of the Child and the principles of social justice, which demand that all children should have equal access to effective childhood vaccines, given the potential of vaccines to save lives and to give all children a chance in life [3, 4].

Some factors responsible for the suboptimal vaccination coverage in areas with ongoing poliovirus circulation in Nigeria are suboptimal OPV acceptance due to unmet health needs, low uptake of vaccination in security-compromised and underserved areas, due to threats and inaccessibility to OPV, challenges reaching the underserved communities, and poor program visibility. Indeed, the epidemiology of polio transmission in Nigeria has continuously shown a pattern of isolation in noncompliant, hard-to-reach, and security-compromised areas of 11 states in northern Nigeria where the risk of polio is very high [5–7].

Although children were chronically missed in Nigeria over successive rounds of supplementary immunization activities (SIAs), not much was done on the part of the program to systematically generate sufficient demand for immunization of this group, especially in areas of traditional polio endemicity in the north. Put differently, despite the desire to vaccinate all children and meet the Global Polio Eradication Initiative targets, the effectiveness of recommended strategies for creating a genuine demand for immunization was limited.

Demand-side interventions have been shown by the World Health Organization and others to lead to significant gains in child vaccination coverage in low- and middle-income countries [8–11]. To stimulate the population to request for OPV, demand creation interventions were introduced during the September 2013 polio vaccination campaign following successive recommendations by the Independent Monitoring Board and the Expert Review Committee, which oversee polio eradication activities in Nigeria. The demand creation activities included the provision of attractive benefits (hereafter, “pluses”) during immunization activities, the establishment of health camps, implementation of the nomadic *Ardo* (ie, Fulani community leader) intervention, the Qur'anic schoolteacher package, and increasing media visibility at the state level.

We describe the implementation and evaluation of several activities to create demand for polio vaccination in persistently poor performing areas of northern Nigeria. These demand creation activities were designed to specifically target children in these communities by providing attractive packages that best respond to caregivers concerns and provide easy access to vaccines among eligible children.

## METHODS

### Evaluation Area

We evaluated the contribution of the various demand creation interventions implemented by WHO Nigeria and its partners in support of the Nigerian federal government. The evaluation focused on local government areas (LGAs) in northern Nigeria at very high risk of polio transmission. Risk assessment analyses were conducted to select very high risk LGAs on the basis of confirmation of polioviruses and proximity to areas with active transmission, population immunity, performance of acute flaccid paralysis (AFP) surveillance, performance of polio SIAs (focusing on missed children and noncompliance), and other population-specific factors, such as presence of nomadic or other high-risk seasonal migratory populations, insecurity, or civil unrest [12, 13].

Seventy-seven LGAs in the following 10 northern Nigerian states were selected for demand creation interventions: Bauchi (3 LGAs), Borno (2), Kaduna (12), Kano (30), Katsina (11), Kebbi (3), Niger (3), Sokoto (4), Yobe (5), and Zamfara (4). Within the LGAs, the targets were children <5 years of age, which is the age group targeted by polio SIAs. The interventions were primarily implemented to improve community acceptance and demand for the polio vaccine.

### Interventions

We used a demand creation strategy comprising a blend of the following 7 composite activities and integrated packages that were given with the polio vaccine in high-risk communities to address health, needs and make the polio vaccine intervention attractive. The goal was to further build trust and confidence in immunization among communities.

#### Attractive Pluses

Attractive pluses consisted of a combination of adult and child add-ons intended to make immunization attractive. Pluses were supported by the WHO and distributed following a plan by the respective field offices down to the operational level. There were different types of child pluses made available to attract children 0–24 months old who were under the care of their mothers or caregivers. Mothers and caregivers who presented their children for immunization got a plus with health value, such as soap, detergent, or sugar. Older siblings who brought eligible children for immunization received similar incentives, to motivate them to search for more unvaccinated children in the neighborhood. Pluses distribution was heralded during town announcements about the value of immunizing children against polio and other vaccine-preventable diseases.

#### Road Shows

Dramatic road shows were used to create visibility and increase awareness of the program and attract children and caregivers outside noncompliant households for vaccinations in the street. The targets were also mothers who were convinced of the benefits of OPV immunization but negatively influenced by either their husband's opinion and decisions or the child's grandmother's convictions not to vaccinate. Dramatic performances involving local theatres were staged during the road shows to stimulate noncompliant mothers into changing their behavior. Shows depicted individuals with permanent disabilities or featured polio survivors themselves, to evoke positive behavioral change, as caregivers witnessed firsthand that their children were at risk of permanent paralysis with continued noncompliance.

#### Qur'anic Teacher Package

Teachers in Qur'anic schools were identified and their location mapped in all high-risk settlements, and a list containing these data was filed at the LGA level. These teachers were influential in passing important religious and social messages (including those related to health) to children and families. Teachers are a traditionally trusted source of information in communities in Nigeria. The package sought to keep identified Qur'anic teachers informed about the polio eradication initiative (PEI) and routine immunization activities in their respective LGAs. They were equipped with answers to frequently asked questions about polio and immunization, communication materials, key messages, fact sheets, and audiovisual materials (ie, CDs/DVDs) for community dialogues on the importance of polio eradication.

#### Nomadic *Ardo* Intervention for Underserved Communities

The *Ardo* intervention was designed for leaders in Fulani sociocultural settings to address the challenges of poor access and acceptability of vaccines among nomads. We contacted *Ardos*, who knew the migratory patterns of their communities and were able to mobilize heads of households in hamlets, to provide dates and time of immunization visits. The *Ardos* also acted as liaisons between the service providers and the communities by reporting communities in which daily implementation of SIA microplans or visits by vaccination teams were not conducted. One *Ardo* per ward was identified and trained to pass the key PEI messages. Meetings were held to provide feedback to the *Ardos* at the end of each immunization exercise. Lessons learned for improving future planning were also documented.

#### Accessing Unreached Children in Security-Challenged Areas

Encouraged by the growing levels of involvement of traditional, religious, and other community leaders in security-compromised areas, the program mapped the locations of influential leaders to enable vaccination teams to administer OPV during campaigns or so-called windows of opportunity. Local heads of religious sects, influential community leaders, youth groups, community-based organizations, and nongovernmental organizations were identified and trained on OPV administration, data recording, and interpersonal communication skills. The selected heads of the group were assigned to mobilize communities, distribute fliers and posters, and even vaccinate children aged <5 years.

#### Health Camps

Communities demanded government and PEI partners to address health needs other than those associated with polio vaccination. In these situations, perception that basic needs were not being provided manifested as noncompliance with polio vaccination, and no communication-related interventions had been effective. An integrated delivery of services in health camps was adopted to address the perceived health needs through provision of free services for minor ailments, with the goal of improving uptake of OPV and other routinely administered vaccines. To efficiently provide services at the health camps, 3 booths were established: (1) OPV administration, (2) routine immunization, and (3) treatment of minor ailments. Community members were informed of the availability of free services through engaged town announcers and religious and community leaders.

#### State-Level Interventions

Efforts were made by state governments to create visibility of PEI activities, through press releases, panel discussions broadcast via radio and television, and jingles. Before launching of polio SIA rounds, states disseminated messages via these outlets about the importance of accepting OPV, to increase awareness of the activities and create demand for immunization services. Furthermore, breakfast meetings were organized for journalists and media producers. This activity served to mobilize resources and create awareness of upcoming immunization activities. During the breakfast meetings, presentations were made on the status of the PEI, after which residual challenges were discussed. Media chief executives then provided support for the realization of the communication targets.

### Data Collection and Analysis

Data on numbers of children reached by different interventions were collected using vaccination team tally sheets, immunization registers, and treatment records in clinic registers. Supervisors measured the quality of implementation of the intervention, using a standardized checklist. Independent monitors provided data on the effectiveness of the intervention against noncompliance with polio SIAs. The data were collated and analyzed with comparison of performance indicators before and after implementation of the interventions. Additionally, AFP surveillance data were used to estimate population immunity in the targeted communities. OPV doses of non–polio-associated AFP cases were analyzed to determine trends before and after implementation of activities, as a proxy for immunity.

## RESULTS

**Figure 1.**
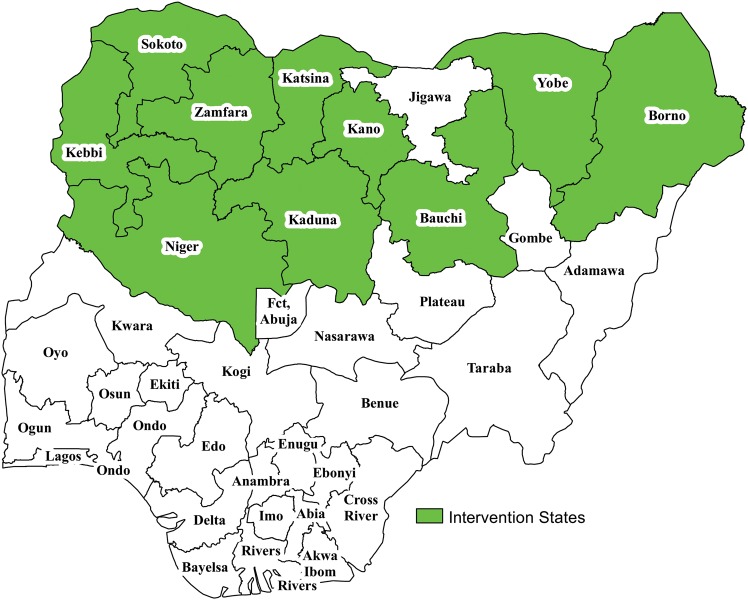
Geographical location of states for demand creation interventions—Northern Nigeria, 2013–2014.

The selected LGAs for demand creation interventions were located in 10 northern states determined to be at very high risk for polio transmission. Figure 1 shows the geographical distribution of the 77 very high-risk LGAs where demand creation activities were implemented, based on risk assessment activities. Kano State had the highest number of LGAs (30), while Kebbi, Niger, and Borno states had the lowest number (4, 3, and 2, respectively). In some states, there was geographical clustering of the LGAs, with the majority of LGAs located on the borders between states or international borders.

There was an increasing trend in the number of children reached by different demand creation interventions from the time activities were introduced in September 2013 to November 2014. Table 1 shows that, among the packages, health camps contributed the largest number of vaccinated children. The highest number of children vaccinated by using this strategy was 731 050 in May 2014, compared with 127 393 children in November 2013. However, the number declined in November 2013, when 668 863 children were vaccinated.

**Table 1.** Trend in the Number of Children Aged <5 Years Immunized in Poor-Performing Local Government Areas, by Intervention Strategy and Supplementary Immunization Activity (SIA) Date—Northern Nigeria, November 2013–November 2014

SIA Date	Health Camps	*Ardo* Interventions	Qur'anic Schools	Road Shows	Total
November 2013	127 393	21 643	71 266	673	220 975
December 2013	154 383	37 372	89 414	4425	285 594
January 2014	239 162	57 799	100 933	11 534	409 428
March 2014	410 105	81 559	134 705	22 015	648 384
April 2014	454 403	64 818	150 371	32 278	701 870
May 2014	731 050	98 127	156 302	52 613	1 038 092
June 2014	691 539	96 601	126 606	33 976	948 722
August 2014	681 842	68 962	119 599	36 153	906 556
September 2014	661 107	217 383	115 533	67 275	1 061 298
November 2014	668 863	74 554	123 545	49 579	916 541
Total	4 819 847	818 818	1 188 274	310 521	7 137 460

Similarly, there was an overall increase in the number of children reached with the *Ardo* intervention. Initially, in November 2013, 21 643 children were vaccinated. The number peaked in September 2014, with 217 383 children vaccinated, but declined in November 2014. Similar increases, with variations, were observed with the Qur'anic schoolteacher package, which recorded 71 266 vaccinated children in November 2013 and 156 302 vaccinated children in May 2014, but there was a decline in November 2014, with 123 545 children vaccinated.

For the road shows intervention, a steady increase in the number of children vaccinated was observed, with the highest number, 67 275 children, vaccinated in September 2014, compared with 696 children in November 2013.

In all, 7 137 460 children were vaccinated through demand creation interventions from November 2013 to November 2014.

**Figure 2.**
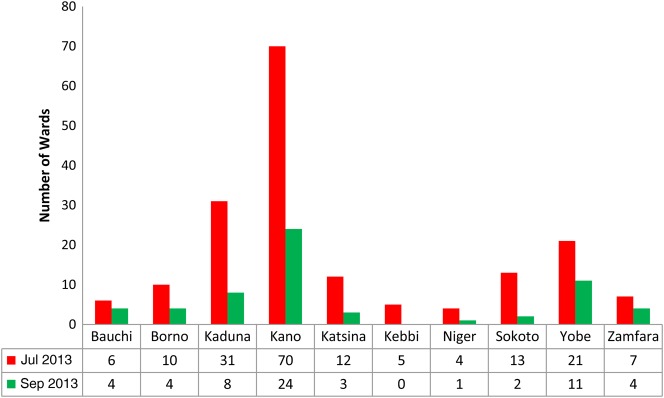
Reduction in the number of wards in poor-performing local government areas with >10% of children missed by supplementary immunization activities due to noncompliance—northern Nigeria, July and September 2013.

An increasing trend in the number of children who received OPV in the persistently poor-performing LGAs further closed the gap of eligible children missed due to noncompliance. Figure 2 illustrates a decline in the number of wards with >10% missed children due to noncompliance across the states that implemented demand creation activities in November 2013, compared with the previous round, conducted in July 2013. The LGAs with the largest decrease in the number of wards with noncompliance were in Kebbi State (from 5 to 0 wards), Sokoto State (from 13 to 2 wards), Kaduna State (from 31 to 8 wards), and Katsina (from 12 to 3 wards).

In terms of population immunity, use of the number of OPV doses received by children with non–polio-associated AFP in targeted LGAs as a proxy revealed an increase in the number of states in which >90% of such children received ≥4 OPV doses, from 2 states in 2012 to 9 states in 2014 (Table 2). The highest proportional increases in the number of children with non–polio-associated AFP who received ≥4 OPV doses between 2012 and 2014 were in states that originally had the lowest proportions of such individuals who received ≥4 doses. For example, the proportion in Sokoto State increased from 43% to 97% (an increase of >2-fold), the proportion in Zamfara State increased from 52% to 100%, and the proportion in Yobe State increased from 63% to 96%. After the introduction of demand creation activities, there was also a steady decline in the number of states in which ≥5% of children with non–polio-associated AFP received 0 doses, from 8 states in 2012 to 0 states in 2014. The largest decline was in Sokoto State, where 11% of children with non–polio-associated AFP received 0 OPV doses in 2012, compared with 0% in 2014.

**Table 2.** Trend in the Number of Oral Polio Vaccine Doses Received by Children With Non–Polio-Associated Acute Flaccid Paralysis (AFP) in Local Government Areas Targeted by Demand Creation Interventions—Northern Nigeria, 2012–2014

	2012			2013			2014		
State	0 doses	1–3 doses	≥4 doses	0 doses	1–3 doses	≥4 doses	0 doses	1–3 doses	≥4 doses
Bauchi	5	30	65	0	22	78	0	9	91
Borno	9	18	73	40	50	10	3	10	87
Kaduna	5	25	70	1	9	90	1	8	91
Kano	6	25	69	2	17	80	0	5	94
Katsina	8	19	73	2	5	93	1	1	99
Kebbi	0	6	94	0	0	100	0	2	98
Niger	0	0	100	0	0	100	0	0	100
Sokoto	11	46	43	0	19	81	0	3	97
Yobe	3	33	63	0	28	72	0	4	96
Zamfara	10	38	52	0	1	99	0	0	100
Overall^a^	3 (56)	17 (357)	80 (1641)	1 (30)	8 (208)	91 (2280)	1 (13)	4 (75)	95 (1778)

Data are % of children with non–polio-associated AFP, unless otherwise indicated.

^a^ Data are overall % (no.) of children with non–polio-associated AFP.

Furthermore, there was a decline in the number of states in which >10% of children with non–polio-associated AFP were underimmunized (defined as those who received 1–3 OPV doses), from >10% in 8 states during 2012 to 0 states having more than 10% under-immunized children by 2014.

Overall, in terms of population immunity, there was an increase in proportion of children with no–polio-associated AFP who received ≥4 OPV doses, from 80% in 2012 to 97% in 2014. There were also decreases between 2012 and 2014 in the proportions of such children who were underimmunized (from 17% to 4%) or received 0 OPV doses (from 3% to 1%).

## DISCUSSION

We found that the 7 demand creation interventions that we implemented in September 2013 increased the uptake of polio vaccines in persistently poor-performing high-risk communities of northern Nigeria. We also found a remarkable reduction in noncompliant households in select LGAs where the demand creation activities were implemented.

The persistently poor-performing LGAs were clustered across the states, which implied that the communities shared the same beliefs in terms of the perceptions to immunization. This corroborates with findings that showed that groups and communities with a common identity and interest behave in similar patterns [14].

Most LGAs were located on the states' borders, which made them more likely to be neglected in terms of provision of basic amenities, including healthcare services, given their distance from the state capital. Also, noncompliance increased in urban areas with rapidly growing populations, where access to health and other social services became limited and the states could not adequately provide the health needs of the increasing populace [15].

We established that there was an increasing trend in the number of children reached by different interventions over 10 SIA rounds when demand creation activities were implemented, especially the health camps. This finding corroborates findings from earlier studies that showed that demand-side interventions led to an increase in child vaccination coverage in diverse low- and middle-income settings and among communities in which, like our study setting, health and social indicators are below standard [9].

It is possible that the health camps reached more children than other interventions because they provided health interventions and other attractive commodities, such as pluses. With the observation that health camps solely supported by the WHO in September 2013 were reaching more children in noncompliant communities, there was immediate buy-in, with a lot of partners supporting the scaling-up of the strategy in the poor-performing LGAs. In this regard, other partners, such as the United Nations Children's Fund and the Bill and Melinda Gates Foundation, funded the design, procurement, and distribution of special health camp kits. Standard operating procedures and guidelines for monitoring were also developed, to improve the efficiency of the health camps.

The involvement of *Ardos* in mapping and planning could have been the reason for the increasing number of nomadic children reached over time. Also, there was deliberate strategic provision of pluses targeting the Fulani population, which could have enhanced interest in vaccination [16]. However, coverage varied in these communities, perhaps because of seasonal migration, during which community members relocated to other states that were not covered by the demand creation interventions, in search of pasture for their animals.

Sensitization of the Qur'anic teachers and equipping them with polio and immunization materials, key messages, and fact sheets improved the accessibility of information on the benefits of immunization and also explained the risks of polio disease. With the expansion of the Qur'anic schoolteacher package, the program was able to deploy more teams to Qur'anic schools, to plan vaccination activities and administer vaccine to children. It also helped dispel beliefs and perceptions that polio vaccination was aimed at harming children and causing infertility. The variations in the number of children reached by this strategy could be attributed to the school calendar.

The presence of other influential community members, such as the group of polio survivors who participated in road shows, put a human face on polio and evoked the intended positive behavior, as caregivers witnessed firsthand that their children at risk for paralysis with continued noncompliance. The intervention also underwent several modifications to make it more entertaining, with the addition of local jesters to draw children to immunization points outside noncompliant households. The road shows were also expanded to include transit sites, such as markets and motor parks, and special occasions, like marriage and naming ceremonies.

The demand creation interventions may have led to an improvement in population immunity. The children who were chronically missed by the program would have been a reservoir for continued polio transmission. Demand creation interventions in Borno State did not have the same impact as in other states, possibly because of other factors that hindered children from being reached.

The increase in acceptance of OPV in previously poor-performing areas drew interest from government and Global Polio Eradication Initiative partners to further invest and scale up the interventions to other areas beyond the initial 77 LGAs, with the aim of increasing population immunity in other vulnerable LGAs in northern Nigeria. It is also worth noting that the provision of treatments for minor ailments assisted in building trust in communities receiving public healthcare interventions, which increased acceptance of OPV.

Despite improvements of demand creation, we recognize some limitations of our approach. First, this report has shown that demand creation interventions cannot alone solve the problem of noncompliance, as their implementation may face barriers such as insecurity and religion. Furthermore, while the interventions were implemented across several states in northern Nigeria, the LGAs involved in the study were not randomly selected, and there was no control to determine whether program performance metrics increased in LGAs that did not implement demand creation activities. Additionally the demand creation interventions were implemented over a relatively short period, which makes it hard to demonstrate their long-term impact. Although the intervention had operating guidelines, with good impact on the program, the execution may not have been consistent in all selected LGAs, and therefore the full potential of the interventions may not have been revealed.

In conclusion, demand creation interventions played a critical role in improving the quality of polio SIAs in communities noncompliant with uptake of polio vaccines and contributed to a reduction in polio transmission. Furthermore, it is important to note that demand creation activities, although specific in this setting to polio eradication, could also be used to improve the success in implementing other public health interventions that face resistance.
